# A nomogram predicting distant metastasis risk for gastric cancer patients with preoperative anemia: a multicenter retrospective study

**DOI:** 10.1186/s12957-024-03486-3

**Published:** 2024-08-01

**Authors:** Guofei Deng, Bo Bi, Huachu Deng, Jingyuan Fan, Zhijian Huang, Changhua Zhang, Yulong He

**Affiliations:** 1 Digestive Diseases Center, The Seventh Affiliated Hospital of Sun Yat-sen University, Shenzhen, 518000 China; 2 Guangdong Provincial Key Laboratory of Digestive Cancer Research, The Seventh Affiliated Hospital of Sun Yat-sen University, Shenzhen, 518000 China; 3 Gastrointestinal Surgery Center, The First Affiliated Hospital of Sun Yat-sen University, Guangzhou, 510080 China; 4 Department of Gastrointestinal and Gland Surgery, the First Affiliated Hospital of Guangxi Medical University, Nanning, 530021 China; 5 Department of Microsurgery, Orthopedic Trauma and Hand Surgery, The First Affiliated Hospital of Sun Yat-sen University, Guangzhou, 510080 China

**Keywords:** Gastric cancer, Distant metastasis, Anemia, Nomogram, Prognosis

## Abstract

**Background:**

Anemia represents a well-established risk factor for patients diagnosed with gastric cancer, and is often associated with an unfavorable prognosis. In this context, the timely prediction of distant metastasis risk in patients with anemic gastric cancer assumes paramount importance.

**Methods:**

Information of gastric cancer patients complicated with preoperative anemia in the First Affiliated Hospital of Sun Yat-sen University was collected. The cohort from the First Affiliated Hospital of Guangxi Medical University was used as an external validation set. A Nomogram was established based on the risk factors screened by univariate and multivariate logistic regression analyses.

**Results:**

A total of 848 gastric cancer patients with preoperative anemia were enrolled. Pyloric obstruction, carcinoma antigen 125, T stage, N stage, tumor size, and preoperative weight loss were independent predictors of distant metastasis in gastric cancer patients with anemia (*p* < 0.05), based on which a nomogram was constructed. The accuracy, reliability and clinical value of the nomogram were evaluated by concordance index, receiver operating characteristic curve, decision curve analysis, calibration curve and showed good stability and clinical predictive value.

**Conclusions:**

Preoperative anemic gastric cancer patients, complicated with pyloric obstruction, elevated CA125, advanced T and N stage, larger tumor size, and preoperative weight loss, should be paid more attention to distant metastasis.

## Introduction

Gastric cancer is a global burden on human health and medical expenditure, ranking fifth as the most common malignant tumor and fourth in terms of mortality rate [1, 2]. Distant metastasis is one of its most malicious clinical phenotypes, which has an immense impact on the prognosis of patients [3, 4]. By providing early identification and prediction of distant metastasis, clinicians can formulate diagnosis plans and strive for improved treatment outcomes and longer survival times.

As a frequent hematological abnormality in many cancers, anemia is highly varied both by cancer type and disease severity [5, 6]. Preoperative anemia in gastric cancer patients has been associated with poor prognosis [7, 8]. Studies have found that anemia can contribute to lower survival rates [9]. Moreover, anemia caused by symptoms of weakness and discomfort reduces the effectiveness of chemotherapy and radiation therapy [10]. Furthermore, anemia resulting in tumor hypoxia caused by depriving tumor cells of oxygen essential for the cytotoxic activity of these drugs can lead to ionizing radiation and chemotherapy resistance, thus reducing tumor sensitivity to radiation and chemotherapy. This, in turn, can result in increased invasiveness and metastatic potential, loss of apoptosis and chaotic angiogenesis, thus further increasing treatment resistance [11]. However, few studies have focused on the risk factors affecting prognosis and adverse outcomes in patients with anemic gastric cancer. Therefore, it is essential to carefully evaluate and predict the prognostic factors for gastric cancer patients with anemia, particularly concerning the risk of distant metastasis, in order to ensure optimal treatment decisions and favorable overall prognosis.

Our research team had previously identified anemia as an independent prognostic factor for non-hypoalbuminemia gastric cancer patients who underwent radical gastrectomy [12]. To better predict the risk of distant metastasis in preoperative anemic gastric cancer patients, we developed a nomogram model based on screening risk factors associated with distant metastasis. This model calculates the risk score and provides a basis for monitoring and predicting distant metastasis of anemic gastric cancer patients.

## Patients and methods

### Inclusion and exclusion criteria

This study included gastric cancer patients who underwent surgery at Sun Yat-sen University’s (SYSU) First Affiliated Hospital between January 2008 and November 2017. Admission criteria included: (1) diagnosed with “gastric cancer” and “anemia” before surgery; (2) not receiving any anti-tumor therapy before surgery; (3) no malignant tumors associated with other organs or systems. Exclusion criteria included: (1) patients who underwent secondary surgery; (2) patients with hematologic disorders; (3) patients who received a blood transfusion in preparation for surgery; (4) patients with missing variables or loss of follow-up. The external validation set of this study was comprised of patients from Guangxi Medical University’s (GXMU) First Affiliated Hospital from May 2017 to January 2020. This study was conducted in line with the Declaration of Helsinki and informed consent had been signed by all patients to collect data for scientific purposes.

### Definition

According to the definition of World Health Organization (WHO) (http://www.who.int/vmnis/indicators/haemoglobin.pdf), in non-pregnant women, anemia is defined by hemoglobin (Hb) level of 120 g/L and in men by 130 g/L. Distant metastasis means malignant tumor cells spread from one primary site to another via lymphatic channels, blood vessels, and body cavities. Clinical symptoms, imaging, intraoperative conditions, postoperative pathological results and follow-up data determine whether a patient has distant metastasis [13, 14].

### Clinical data collection and processing

The collected data included general information (age, gender, Body Mass Index (BMI), smoking history, alcohol use, American Society of Anesthesiologists (ASA) physical status score), clinical symptoms (pyloric obstruction, weight loss, hypertension, diabetes, coronary heart disease (CHD)), laboratory inspection (White blood cell count (WBC), Neutrophil (NEU), platelets (PLT), albumin (ALB), alpha-fetoprotein (AFP), carcino-embryonic antigen (CEA), carbohydrate antigen 125 (CA125)), oncologic features (primary tumor location, tumor size, T stage, N stage, tumor TNM stage, occurrence of metastasis) and follow-up status. The information above was recorded into a baseline table for subsequent analysis.

### Follow-up

All the enrolled patients were routinely followed up by special personnel, and the frequency of follow-up gradually changed from 6 months to 1 year until death. The last follow-up date was May 2018 in the SYSU cohort and November 2022 in the GXMU cohort. The study endpoint event was overall survival (OS), calculated from the surgery date to the time of the endpoint event or the date of the last follow-up. To assess the survival status of the patients and their general situation, telephone and E-mail was the primary form of follow-up.

### Establishment and validation of the nomogram

A nomogram was developed utilizing the independent prognostic factors of distant metastasis in gastric cancer patients with anemia. The discriminant ability of the nomogram was estimated using the Concordance Index (C-Index), and area under the receiver operating characteristic (ROC) curve analysis was conducted to further assess the predictive ability of the nomogram [15]. The optimal cut-off point was identified based on the Youden index [16]. The C-index and area under the curve (AUC) values range from 0.5 to 1.0, with 0.5 representing random chance and 1.0 indicating a perfect fit. Generally, C-index and AUC values greater than 0.7 are indicative of a satisfactory estimation. Next, a decision curve analysis (DCA) was conducted to assess the clinical effectiveness of the model [17], and a calibration curve was created to illustrate the difference between the actual results and the predicted value [18]. Finally, the net reclassification index (NRI) and integrated discrimination improvement (IDI) were employed to assess the clinical benefits and utility of the nomogram in comparison to the independent factors [19, 20]. These two metrics serve as alternatives to AUC for evaluating the enhancement in risk prediction and gauging the usefulness of a new model.

### Assessment of the nomogram

In order to evaluate the clinical utility of the nomogram, the risk score of each patient was calculated according to the nomogram and the patients were divided into high- and low-risk groups based on the median score. The time-dependent receiver operating characteristic curve (time-ROC) was utilized to assess the discriminative capacity in forecasting 1-, 3-, and 5-year prognosis, while a Kaplan-Meier curve was employed to compare the disparity between different risk groups. The incidence of distant metastasis in different risk groups was also compared. Finally, we performed a clinical correlation analysis between risk score and clinical features, and the results were presented using boxplots. Statistical significance was set at *p* < 0.05 for both sides.

### Statistical analysis

Categorical variables were summarized as percentages and analyzed using the Chi-square test or Fisher’s exact test, while continuous variables were expressed as mean with standard deviation or interquartile range. Univariate regression analysis was used to identify potential predictors, which were then included in multivariate regression analysis for further evaluation. Subsequently, independent predictors associated with distant metastasis were identified. *p* < 0.05 was used as the criteria for inclusion and exclusion. Statistical analyses were conducted with “rms” package, “pROC” package, “rmda” package, “survival” package, and “limma” package of R version 4.1.1.

## Results

### Clinical characteristics of the patients

**Fig. 1:**
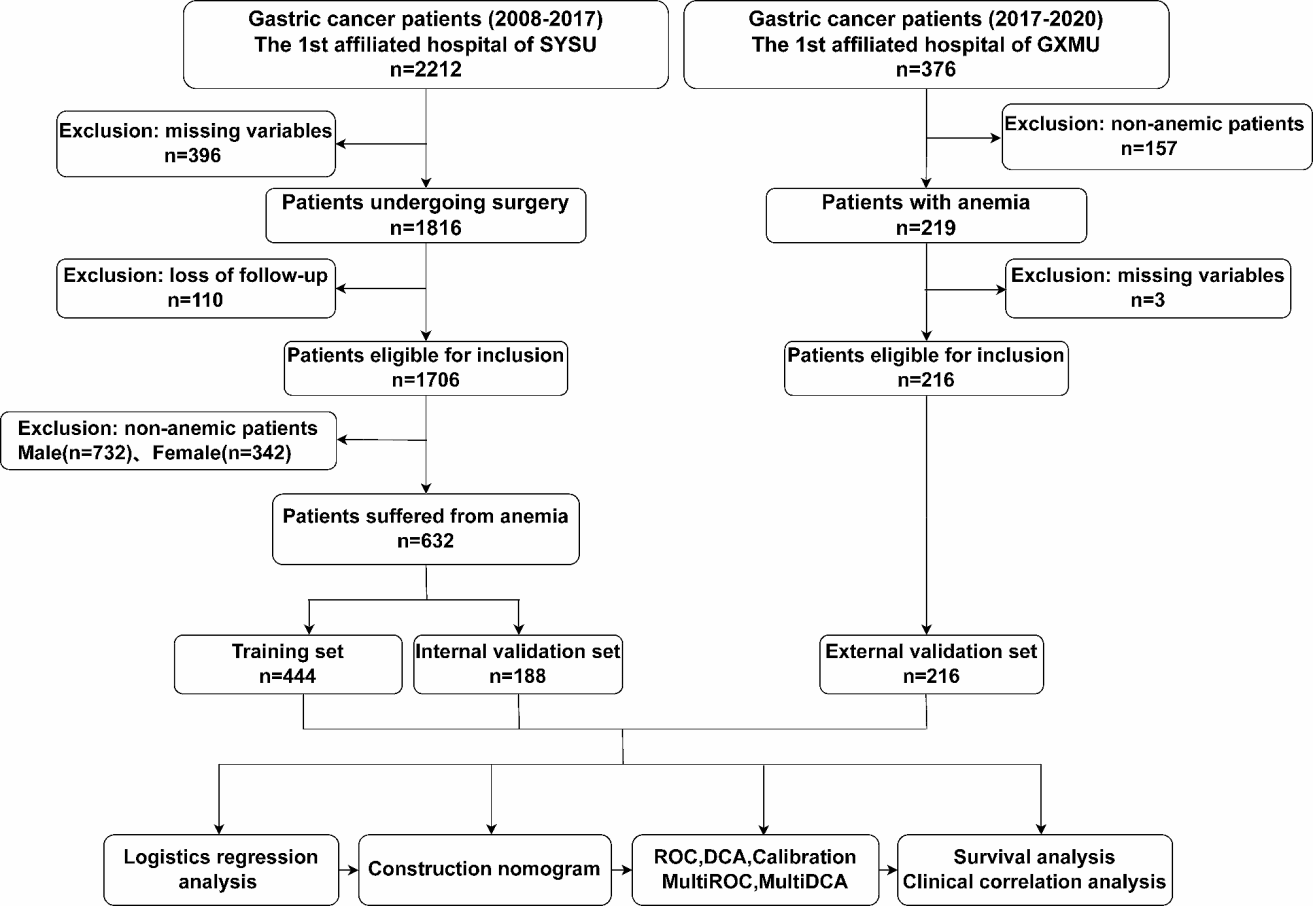
The flowchart of the study

**Fig. 2:**
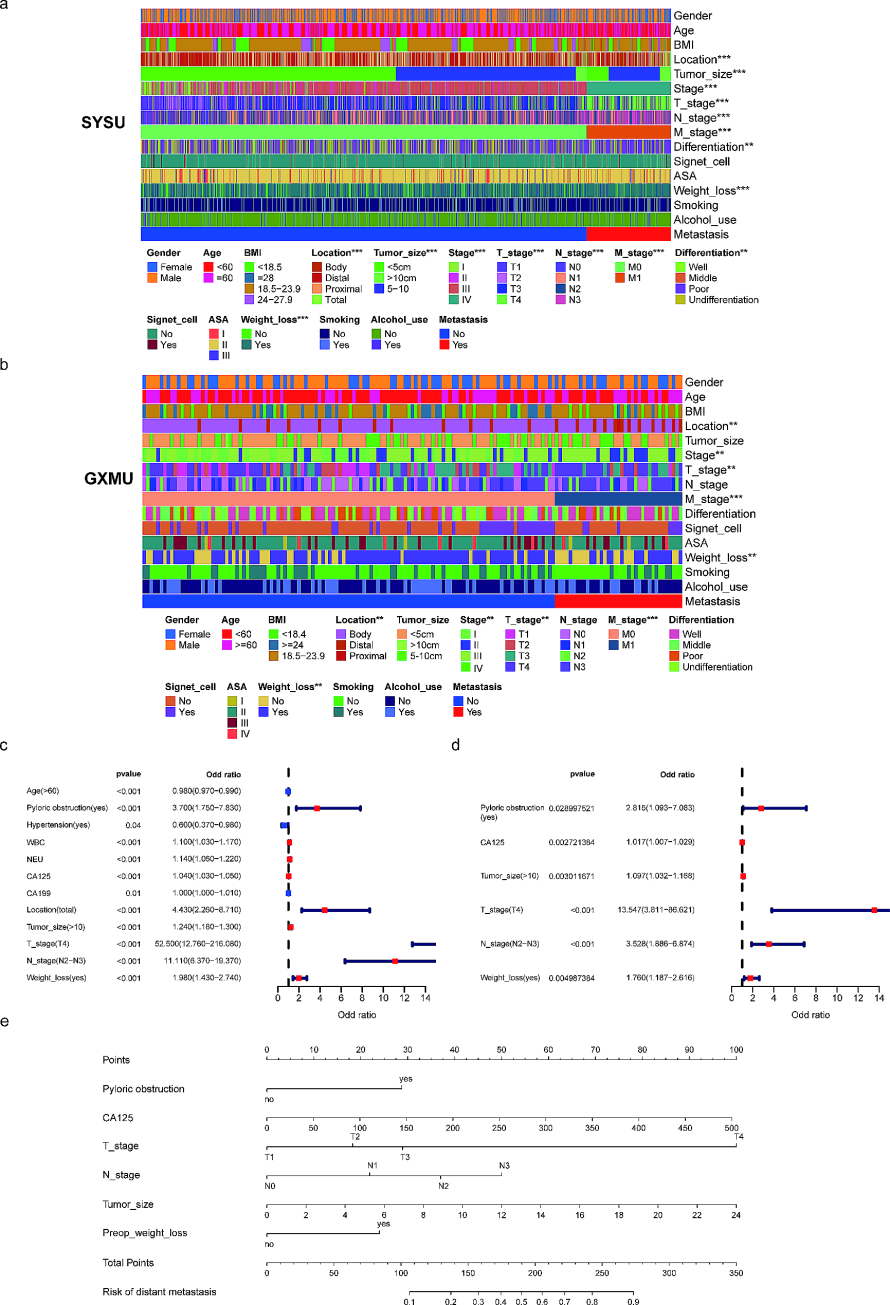
Clinical features of the Sun Yat-sen University (SYSU) cohort (**a**) and Guangxi Medical University (GXMU) cohort (**b**). The forest plot of the univariate (**c**) and multivariate logistics analysis (**d**). The nomogram to predict distant metastasis risk for anemic gastric cancer patients (**e**)

The total number of patients studied was 848. Of these, 632 eligible patients from SYSU cohort were enrolled in this study, which were divided into a training set and an internal validation set on a 7:3 basis. 216 patients from GXMU cohort were regarded as the external validation set. The process of screening patients was shown in Fig. 1. The characteristics of all enrolled patients were illustrated in Fig. 2a and b. More details about patients in different groups were presented in Table 1.

**Table 1:** Clinical characteristics of gastric cancer patients

Variables		level	Training set	Internal validation	External validation
			(*n* = 444)	(*n* = 188)	(*n* = 216)
Gender (%)					
		Male	285 (64.2)	114 (60.6)	133 (61.6)
		Female	159 (35.8)	74 (39.4)	83 (38.4)
Age (mean (SD))			59.14 (11.99)	60.52 (13.35)	54.39 (13.13)
BMI (mean (SD))			19.24 (6.83)	18.63 (7.62)	21.25 (3.36)
Weight loss (%)		No	182 (41.0)	72 (38.3)	64 (29.6)
		Yes	262 (59.0)	116 (61.7)	152 (70.4)
Smoking (%)		No	370 (83.3)	158 (84.0)	150 (69%)
		Yes﻿	74 (16.7)	30 (16.0)	66 (31%)
Alcohol use (%)		No	382 (86.0)	166 (88.3)	135 (62%)
		Yes	62 (14.0)	22 (11.7)	81(38%)
PO (%)		No	428 (96.4)	181 (96.3)	181 (83.8)
		Yes	16 (3.6)	7 (3.7)	35 (16.2)
Hypertension (%)		No	368 (82.9)	144 (76.6)	191 (88.4)
		Yes	76 (17.1)	44 (23.4)	25 (11.6)
Diabetes (%)No			407 (91.7)	171 (91.0)	205 (94.9)
Yes			37 (8.3)	17 (9.0)	11 (5.1)
CHD (%) No			428 (96.4)	181 (96.3)	205 (94.9)
Yes			16 (3.6)	7 (3.7)	11 (5.1)
COPD (%)		No	442 (99.5)	186 (98.9)	216 (100)
		Yes	2 (0.5)	2 (1.1)	0 (0)
ASA (%) I			49 (11.0)	16 (8.5)	12 (4.3)
II			360 (81.1)	161 (85.6)	150 (70.4)
III			33 (7.4)	11 (5.9)	45 (21.1)
IV			2 (0.5)	0 (0.0)	9 (4.2)
WBC (mean (SD))			6.13 (2.14)	6.16 (3.19)	4.62 (1.02)
NEU (mean (SD))			3.86 (2.17)	3.73 (1.94)	3.61 (1.69)
PLT (mean (SD))			275.43(108.97)	260.39 (102.83)	232.01 (77.16)
ALB (mean (SD))			36.56 (5.60)	36.89 (5.01)	36.49 (3.78)
AFP (median [IQR])			2.54[1.81,3.71]	2.58 [1.82, 3.96]	2.46 [1.75, 4.02]
CEA (median [IQR])			2.15[1.21,4.00]	2.12 [1.22, 3.75]	1.86 [1.17, 3.66]
CA125 (median [IQR])			11.10[7.30,16.52]	10.35[6.57,16.45]	10.00 [7.30, 14.45]
Location (%)	proximal		128 (28.8)	59 (31.4)	9 (4.2)
	body		113 (25.5)	47 (25.0)	179 (82.9)
	distal		183 (41.2)	78 (41.5)	28 (13.0)
	total		20 (4.5)	4 (2.1)	0 (0)
Tumor size (mean (SD))			5.45 (3.11)	5.14 (3.17)	4.33 (2.13)
Differentiation (%) well			7 (1.6)	3 (1.6)	82 (38.0)
middle			109 (24.5)	32 (17.0)	40 (18.5)
poor			260 (58.6)	121 (64.4)	34 (15.7)
undifferentiation			68 (15.3)	32 (17.0)	60 (27.8)
Signet_cell (%) No			413 (93)	170 (90.4)	161 (74.5)
Yes			31 (7.0)	18 (9.6)	55 (25.5)
Stage (%)		I	40 (9.0)	20 (10.6)	44 (20.4)
		II	93 (20.9)	34 (18.1)	45 (20.8)
		III	225 (50.7)	91 (48.4)	124 (57.4)
		IV	86 (19.4)	43 (22.9)	3 (1.4)
T stage (%)		T1	37 (8.3)	19 (10.1)	34 (15.7)
		T2	45 (10.1)	15 (8.0)	38 (17.6)
		T3	241 (54.3)	98 (52.1)	54 (25.0)
		T4	121 (27.3)	56 (29.8)	90 (41.7)
N stage (%)		N0	119 (26.8)	50 (26.6)	58 (26.9)
		N1	132 (29.7)	47 (25.0)	39 (18.1)
		N2	96 (21.6)	43 (22.9)	35 (16.2)
		N3	97 (21.8)	48 (25.5)	84 (38.9)
Metastasis (%)		No	366 (82.4)	150 (79.8)	179 (82.9)
		Yes	78 (17.6)	38 (20.2)	37 (17.1)

SD: standard deviation, IQR: interquartile range, BMI: body mass index, PO: pyloric obstruction, CHD: coronary heart disease, COPD: chronic obstructive pulmonary disease, ASA: American society of Anesthesiologists (ASA) physical status classification system, WBC: white blood cell, NEU: Neutrophil, PLT: platelets, ALB: albumin, AFP: alpha-fetoprotein, CEA: carcino-embryonic antigen, CA125: carbohydrate antigen 125

### Independent risk factors of distant metastasis

To identify the risk factors of distant metastasis, 29 preoperative variables were included. The results of the univariate and multivariate logistic regression analysis are presented in Table 2. Age, pyloric obstruction, WBC, NEU, CA125, CA199, tumor location, tumor size, T stage, N stage, and preoperative weight loss were strongly associated with distant metastasis according to univariate logistic regression (Fig. 2c, *p* < 0.05). Multivariate analysis identified pyloric obstruction, CA125, T stage, N stage, tumor size and preoperative weight loss as the independent predictors of distant metastasis (Fig. 2d, *p* < 0.05), which were chosen to develop the nomogram.

**Table 2:** Logistic regression analyses of distant metastasis in gastric cancer patients

Variables	Univariate analysis				Multivariate analysis		
	OR	95%CI	*p*		OR	95%CI	*p*
Gender(female)	1.22	0.88–1.70	0.24				
Age( > = 60)	0.98	0.97–0.99	< 0.001		0.99	0.97-1.00	0.10
BMI( > = 24)	0.96	0.89–1.03	0.27				
Pylorochesis(yes)	3.70	1.75–7.83	< 0.001		2.81	1.09–7.08	0.03
Diabetes(yes)	0.43	0.19–1.01	0.05				
Hypertension(yes)	0.60	0.37–0.98	0.04		0.65	0.35–1.17	0.16
CHD(yes)	1.72	0.76–3.88	0.19				
COPD(yes)	0	0–1.00	0.98				
WBC	1.10	1.03–1.17	< 0.001		1.06	1.00-1.18	0.10
NEU	1.14	1.06–1.22	< 0.001		1.04	0.90–1.17	0.54
HCT	0.17	0.02–1.53	0.11				
Blood type(AB)	1.05	0.50–2.19	0.89				
ALB	0.98	0.95-1.00	0.06				
GLB	1.01	0.98–1.05	0.35				
GLU	1.03	0.95–1.11	0.46				
AFP	1.00	1.00-1.01	0.71				
CEA	1.00	1.00-1.01	0.38				
CA125	1.04	1.03–1.05	< 0.001		1.02	1.01–1.03	0.003
CA199	1.00	1.00-1.01	0.01		1.01	0.99-1.00	0.55
Location(total)	4.43	2.26–8.71	< 0.001		1.40	0.60–3.22	0.43
Tumor_size(> 10 cm)	1.24	1.18–1.3	< 0.001		1.10	1.03–1.17	0.003
T_stage(T4)	52.5	12.76-216.08	< 0.001		13.55	3.81–86.62	0.001
N_stage(N3)	11.11	6.37–19.37	< 0.001		3.53	1.89–6.87	0.001
Weight loss(yes)	1.98	1.43–2.74	< 0.001		1.76	1.19–2.62	0.005
Differentiation(poor)	626	0–1.00	0.99				
Signet_cell(yes)	1.42	0.80–2.52	0.23				
ASA	319	0–1.00	0.98				
Smoking history	0.95	0.62–1.46	0.82				
Alcohol use	0.75	0.45–1.27	0.28				

OR: odd ratio, CI: confidence index, BMI: body mass index, CHD: coronary heart disease, COPD: chronic obstructive pulmonary disease, WBC: white blood cell, NEU: Neutrophil, HCT: hematocrit, PLT: platelets, ALB: albumin, GLB: globulin, GLU: glucose, AFP: alpha-fetoprotein, CEA: carcino-embryonic antigen, CA125: carbohydrate antigen 125, CA199: carbohydrate antigen 199, ASA: American society of Anesthesiologists (ASA) physical status classification system

### Construction and validation of the nomogram for distant metastasis

**Fig. 3:**
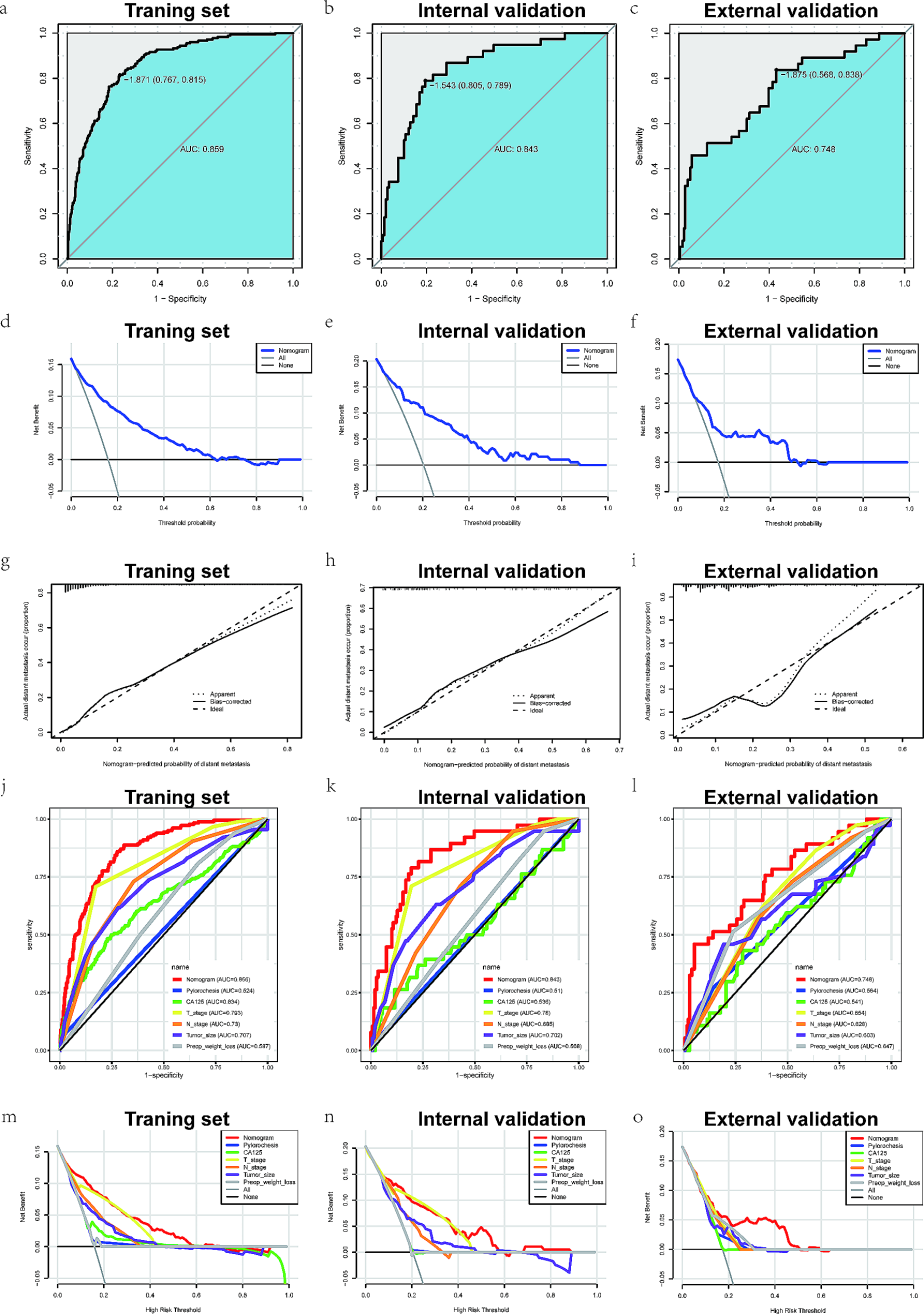
The ROC curve of the training set (**a**), internal validation set (**b**) and external validation set (**c**). The DCA curve of the training set (**d**), internal validation set (**e**) and external validation set (**f**). The calibration curve of the training set (**g**), internal validation set (**h**) and external validation set (**i**). The multiROC curve of the training set (**j**), internal validation set (**k**) and external validation set (**l**). The multiDCA curve of the training set (**m**), internal validation set (**n**) and external validation set (**o**)

A nomogram of distant metastasis was constructed by integrating the six independent predictors above (Fig. 2e). The C-index of the nomogram for identifying distant metastasis in the training set was 0.859 [95% confidence interval (95% CI): 0.831–0.887]. In the validation sets, the C-index was 0.843 (95% CI: 0.774–0.912) internal and 0.748 (95% CI: 0.657–0.839) external. The performance of the nomogram was evaluated by calculating AUC of the ROC curve, with the AUC in training set being 0.859 (sensitivity = 0.767, specificity = 0.815, Fig. 3a), 0.843 (sensitivity = 0.805, specificity = 0.789, Fig. 3b) in the internal validation set, and 0.748 (sensitivity = 0.568, specificity = 0.838, Fig. 3c) in the external validation set. Subsequently, we conducted DCA analyses to evaluate the clinical efficacy of the nomogram, which revealed excellent clinical relevance, as demonstrated in Fig. 3d and f. Moreover, the prediction and the actual incidence of distant metastasis were in good agreement, as evidenced by the calibration curve (Fig. 3g and i). The C-index, NRI, and IDI all demonstrated that the nomogram model was more accurate in predicting performance compared to individual factors (Table 3).

**Table 3:** C-index, NRI, and IDI of the nomogram and predicted factor alone in predicting distant metastasis for gastric cancer patients

Group	Nomogram	Pylorochesis	CA125	T_stage	N_stage	Tumor size	Weight loss
C-index							
Training set	0.859	0.524	0.634	0.793	0.73	0.707	0.587
Internal validation	0.843	0.51	0.536	0.78	0.685	0.702	0.568
External validation	0.748	0.564	0.541	0.654	0.628	0.603	0.647
NRI(vs.Nomogram)							
Training set	-	0.450	1.086	0.025	0.227	0.353	0.482
Internal validation	-	0.504	1.164	0.060	0.317	0.386	0.504
External validation	-	0.357	0.638	0.357	0.357	0.283	0.082
IDI(vs.Nomogram)							
Training set	-	0.258	0.228	0.064	0.169	0.188	0.253
Internal validation	-	0.272	0.274	0.063	0.200	0.196	0.256
External validation	-	0.130	0.148	0.102	0.116	0.120	0.096

NRI: net reclassification index, IDI: integrated discrimination improvement

### Comparison of nomogram with individual independent factors

The predictive effectiveness of the nomogram was further assessed by comparison with individual independent prognostic factors using multiple ROC and DCA curves. The results of the multiple ROC curves in the training set showed that the AUC of the nomogram (AUC = 0.856) was superior to that of the pyloric obstruction (AUC = 0.524), CA125 (AUC = 0.634), T stage (AUC = 0.793), N stage (AUC = 0.730), tumor size (AUC = 0.707), and preoperative weight loss (AUC = 0.587) (Fig. 3j). In the validation sets, the results were consistent with the training set. The AUC values of the multiple ROC curves in the internal validation set were the nomogram (AUC = 0.843), T stage (AUC = 0.78), tumor size (AUC = 0.702), N stage (AUC = 0.685), preoperative weight loss (AUC = 0.568), CA125 (AUC = 0.536), and pyloric obstruction (AUC = 0.51) from high to low, respectively (Fig. 3k). And those in the external validation set were nomogram (AUC = 0.748), pyloric obstruction (AUC = 0.564), CA125 (AUC = 0.541), T stage (AUC = 0.654), N stage (AUC = 0.628), tumor size (AUC = 0.603), and preoperative weight loss (AUC = 0.647) (Fig. 3l). Additionally, multiple DCA curves results indicated that the nomogram model was more reliable than the individual independent prognostic factor in both the training and validation sets (Fig. 3m and o).

### Survival analysis of patients in different risk groups

**Fig. 4:**
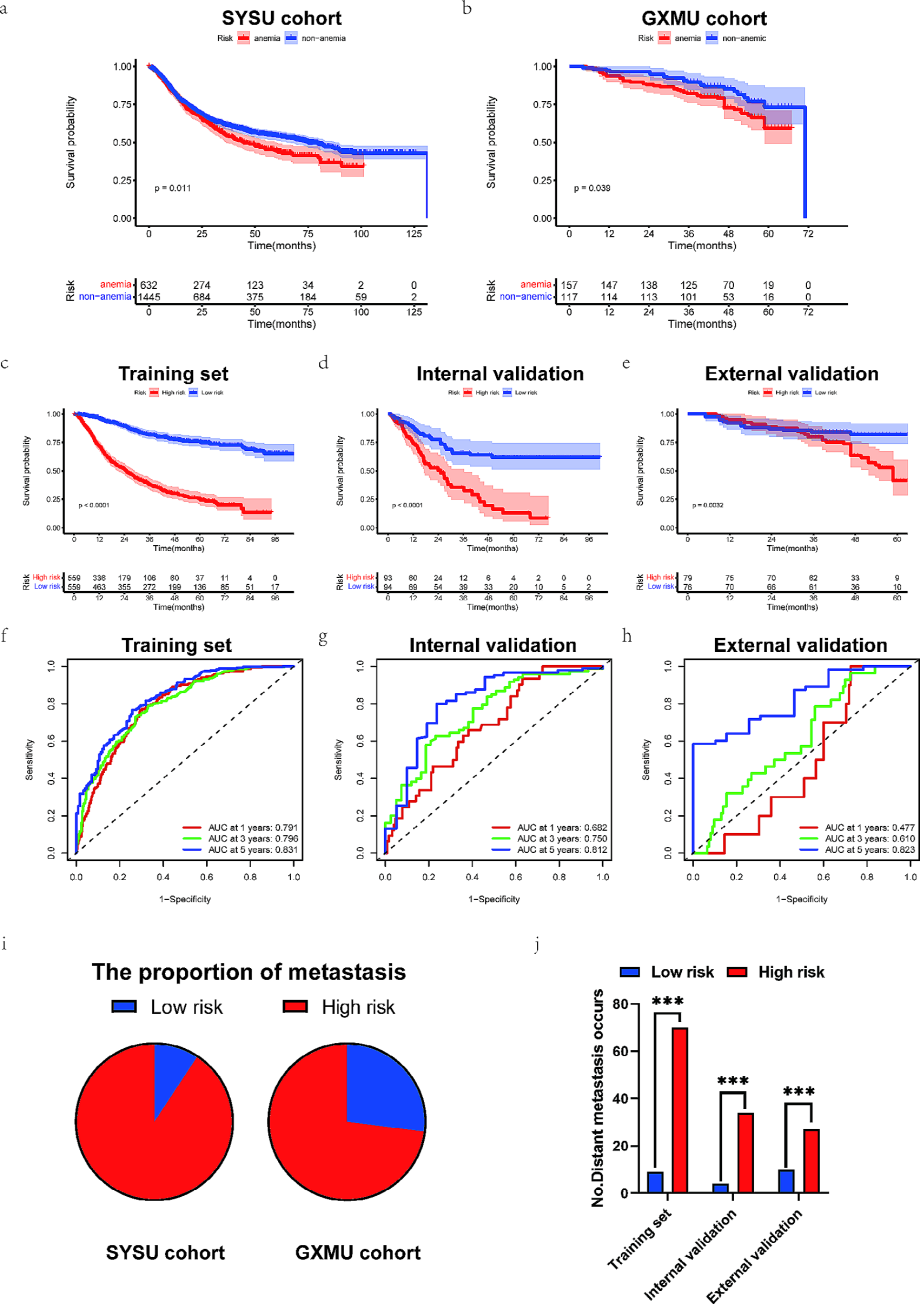
Kaplan–Meier curve for overall survival in the Sun Yat-sen University cohort (**a**) and Guangxi Medical University cohort (**b**) between anemic and non-anemic patients. Kaplan–Meier curve for overall survival in the training set (**c**), internal validation set (**d**) and external validation set (**e**) between low- and high-risk patients. Time-ROC curve for 1-, 3- and 5-year prognosis in the training set (**f**), internal validation set (**g**) and external validation set (**h**). The proportion of metastasis patients in the Sun Yat-sen University cohort and Guangxi Medical University cohort (**i**). Differences in the number of patients with distant metastasis between low- and high-risk groups in different sets (**j**)

We reviewed the initial cohort’s survival differences between anemic and non-anemic patients. Patients with anemia exhibited a poorer prognosis than non-anemic patients in both the SYSU cohort (Fig. 4a) and GXMU cohort (Fig. 4b). The results are consistent with the conclusions of previous studies [7, 12]. Subsequently, the risk score of all patients in the training set was computed based on the nomogram information atlas. Using the median risk score of all patients, we separated the patients into low-risk group (risk score < = 113.3) and high-risk group (risk score > 113.3). The Kaplan-Meier curve effectively demonstrated the association between risk stratification and prognosis, with the prognosis of patients in the high-risk group being poorer than that of the low-risk group (Fig. 4c and e). Additionally, the time-ROC revealed that the model had a robust capacity in predicting prognosis, particularly in predicting long-term prognosis, with AUC values reaching 8 (Fig. 4f and h). The risk score was found to be associated with distant metastasis, and it was observed that the high-risk group had a greater probability of distant metastasis in both the SYSU and GXMU cohorts (Fig. 4i), with statistical significance in each cohort (Fig. 4j).

### Relationship between clinical features and risk score of the nomogram

**Fig. 5:**
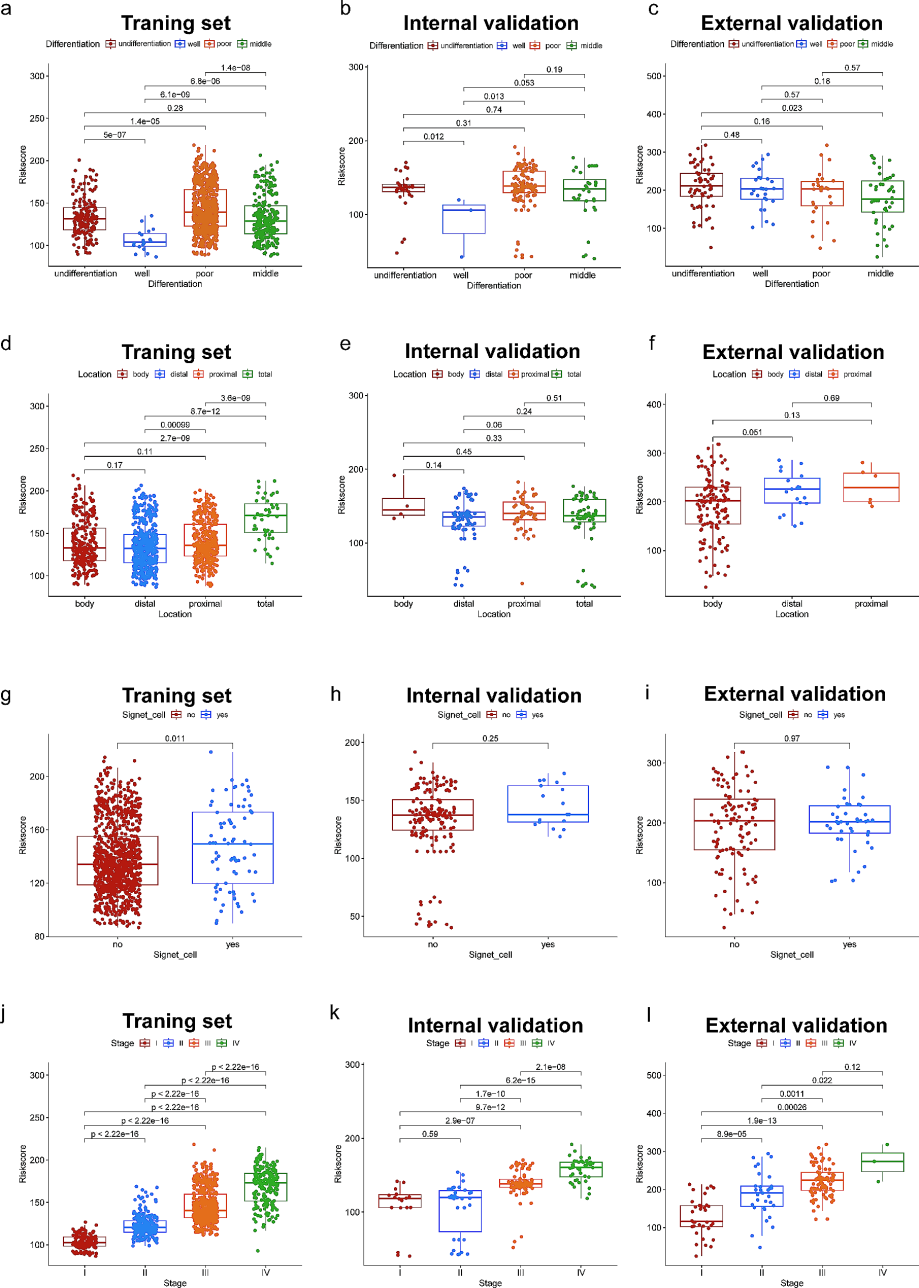
Boxplot of the relationship between risk score and tumor differentiation in the training set (**a**), internal validation set (**b**) and external validation set (**c**). Boxplot of the relationship between risk score and tumor location in the training set (**d**), internal validation set (**e**) and external validation set (**f**). Boxplot of the relationship between risk score and signet cell type in the training set (**g**), internal validation set (**h**) and external validation set (**i**). Boxplot of the relationship between risk score and tumor TNM stage in the training set (**j**), internal validation set (**k**) and external validation set (**l**)

Clinical features of gastric cancer patients with anemia were also collected, including tumor differentiation, location, signet cell type, and stage. Each patient was assigned a risk score according to the nomogram, and a correlation analysis was carried out with the above clinical features. The results showed that tumor differentiation (Fig. 5a and c), location (Fig. 5d and f), signet cell type (Fig. 5g and i), and tumor stage (Fig. 5j and l) were associated with risk score in various groups, with higher risk score indicating worse differentiation types, and severer tumor staging.

## Discussion

In this study, a nomogram model was developed and validated to assess distant metastasis risk among anemic gastric cancer patients and predict their long-term survival, which would help formulate a better clinical evaluation and intervention.

Anemia is common and multifactorial in patients with malignant tumors. Recent studies have focused on anemia in gastric cancer, suggesting that anemia is negatively associated with quality of life and prognosis. Lim et al. found that anemia was predominantly caused by iron deficiency anemia after resection of early gastric cancer [21]. In a 5-year follow-up of patients who underwent gastrectomy, Jun et al. found that the incidence of anemia was higher in women, followed by total gastrectomy, diabetes, and low BMI [9]. Park et al. studied patients undergoing 5-fluorouracil-based chemotherapy in gastric cancer and showed that anemia could be a decisive, independent prognostic factor [22]. In patients with stage I and II gastric cancer, Shen et al. found that anemia led to a poorer prognosis [23]. Huang et al. conducted a meta-analysis of 13,154 patients in 17 studies, and preoperative anemia was also found to predict poor OS and disease-free survival (DFS) of gastric cancer [7]. These studies demonstrated that anemia was closely related to the occurrence and progression of gastric cancer. Distant metastasis is one of the clinical manifestations of most malignant tumors [24]. Due to the insidious onset and atypical symptoms of gastric cancer, distant metastasis may already be present in some patients when they are first diagnosed with gastric cancer [25]. It has been demonstrated that the prognosis of the tumor is significantly correlated with the presence of distant metastasis. Early identification and assessment of distant metastasis can help to formulate more effective response measures and strive for better treatment outcomes for anemic gastric cancer patients. However, most studies only focused on the prognostic impact of anemia on patients with gastric cancer, and few could relate clinical traits to the prognosis of gastric cancer patients with anemia. Therefore, understanding the pathological features of distant metastasis in anemic gastric cancer was of great importance.

Through univariate and multivariate logistic regression analysis, our model incorporated six independent predictors, including pyloric obstruction, CA125, T stage, N stage, tumor size and preoperative weight loss. Pyloric obstruction refers to gastric outlet obstruction, which is manifested as abdominal distention, nausea, vomiting, and ultimately malnutrition. It severely affects the quality of life, reflecting the tumor size and malignancy. Moreover, gastric cancers featuring pyloric obstruction usually display invasive growth patterns and predominantly consist of undifferentiated adenocarcinomas, which frequently lead to a higher occurrence of distant metastasis [26]. CA125 is recognized as a tumor marker for gastrointestinal tumors [27]. It is widely used in clinical practice due to its high sensitivity and specificity, and can be easily obtained by blood sampling [28]. The T stage and N stage adopt the evaluation method of the seventh edition of the American Joint Committee on Cancer (AJCC) to assess the extent of tumor invasion and lymph node metastasis, which have become important tools to guide the clinical staging and treatment of gastric cancer patients. Tumor size has been proved to be a significant risk factor for distance metastasis in ductal carcinoma in situ, metastasis incidence had a direct relationship with tumor size [29]. Gastric cancer patients often experience weight loss before surgery, research has pointed out that weight loss can be a significant predictor of distant metastatic potential and overall prognosis [30]. The six independent predictors included not only the traditional recognized indicators reflecting the malignant degree of gastric cancer (T stage, N stage), but also the widely used laboratory indicators (CA125, tumor size), as well as the common clinical manifestations of gastric cancer patients (pyloric obstruction and preoperative weight loss), which reflected the malignancy of the tumor in different ways and were closely correlated with distant metastasis, indicating that the model has high reliability. And these indicators can be obtained through medical history collection and simple clinical examination in the process of diagnosis and treatment, with strong clinical acquisition and feasibility. Furthermore, multiple ROC and DCA curves showed that the nomogram had better predictive performance than a single independent predictor, as the AUC value of the nomogram was the highest of all the variables.

By plotting the KM survival curve and time-ROC, we found that the nomogram model’s risk score was associated with the prognosis of patients. Using the risk score of the nomogram to group patients into high- and low-risk groups facilitates early recognition of distant metastases and provides a tool for predicting patient outcomes. Further analysis of the correlation between clinical features and the model’s risk score revealed that differentiation, an indicator of tumor malignancy, was also associated with the risk score. The worse the degree of differentiation, the higher the degree of malignancy [31], suggesting that there may also be differences in genetic susceptibility, pathological features, clinical manifestations, and prognosis of anemic gastric cancer patients, which need further subgroup analysis [32].

To better integrate the nomogram into the clinical workflow, we can use these indicators to design a panel or questionnaire to score the risk of distant metastasis in anemic gastric cancer patients. For patients with higher scores, more attention should be paid. It is noteworthy that pyloric obstruction was identified as a risk factor for distant metastasis. This implies that doctors should be aware of the complication of pyloric obstruction, and more aggressive treatment should be considered in those patients with gastric cancer who have anemia and pyloric obstruction simultaneously. Of course, this study has certain limitations: (1) The large time span of the SYSU cohort included in this study, and the past data collection methods, tools, or standards may differ from those of the current, making direct comparison of results difficult. (2) Treatment, diagnostic techniques, and epidemiologic features may change over time, affecting the interpretation and generalizability of findings. (3) Some subjects may have lost contact or no longer participate in the study during the follow-up period, and follow-up information may be missing or incomplete, affecting the accuracy of outcome variables.

In general, this Nomogram model for anemic gastric cancer patients has good accuracy and stability in predicting the risk of distant metastasis, patient prognosis, and clinical features of the tumor, showing great potential for application. Future research could employ Mendelian Randomization and other statistical methods to explore the causal connection between anemia and distant metastasis on a deeper genetic level [33]. We hope to include more samples for further validation in the future and conduct subgroup analysis based on gender, age, tumor stage, and other clinical traits in order to perfect the nomogram prediction model of distant metastasis and provide an accurate and stable evaluation tool for improving the prognosis of patients with anemic gastric cancer.

## Conclusions

The nomogram model strongly predicts the risk of distant metastasis and long-term survival in patients with anemic gastric cancer. Patients with anemic gastric cancer exhibiting combined pyloric obstruction, elevated CA125, advanced T and N stage, larger tumor size, and preoperative weight loss should be cognizant of the heightened risk of developing distant metastases and poor prognosis.

## Data Availability

All data included in this study are available upon request by contact with the corresponding author on reasonable request.
